# Supplementary material for: Quantifying the effects of delisting wolves after the first state began lethal management
Source: PeerJ. 2021 Jul 5;9:e11666. doi: 10.7717/peerj.11666 (PMC8265384; doi:10.7717/peerj.11666)
Supplement: Supplemental Information 2 [file peerj-09-11666-s002.docx]

**Supplementary Material 1: the public and governmental context of the Wisconsin wolf-hunt**

The case we described in main text includes court action, legislative action, executive branch action, and public comment.

**Judicial branch**

In mid-February 2021, a circuit court in Jefferson County issued a writ of mandamus ordering the state wildlife agency to hold the hunt as soon as legally feasible in February 2021. The state appealed to a higher court which declined jurisdiction because the circuit court had not formally closed the case.

**Legislative branch**

In 2012, the Wisconsin legislature passed Act 169 designating gray wolves as game, mandating an annual wolf-hunt to begin in the first week of November each year the wolf is not under federal protection. The unusual statutory mandate to hold a hunt took some authority away from the executive branch in deciding when or if such a hunt should be held. That legislative action informed the subsequent judicial action in 2021 mentioned above.

**The Natural Resources Board (NRB)**

The NRB is overseen by both the legislative and executive branches.

After initially voting against authorizing a wolf-hunt on 22 January 2021 (<https://dnrmedia.wi.gov/main/Play/731c92f70bb84be69b8f69ef1ccbb99c1d> accessed 27 April 2021), the NRB responded to the above court action by approving a wolf-hunt beginning 22 February 2021 with a quota of 200 non-reservation wolves, availability of 20 times that number of permits, and the zonal quotas summing to 200 [1]. In the video-recording of the latter NRB meeting, three topics relevant to our present context were also discussed [2]. All quotations below derive from the latter meeting and the video-recording at that link.

The chair, F. Prehn, added to the official record regarding the judicial proceeding described above. He reported that neither he nor the NRB had been consulted on filing the appeal [2].

NRB members reaffirmed the state population goal and declared that the NRB was not aiming to stabilize the population but rather “Move population toward population goal.” And “The current population is almost four times higher.” [2], which referred to 350 wolves [3-5].

Following a question about science behind the quota of 200, DNR’s D. MacFarland stated, “[DNR] started with the population estimate and there are two published studies by Fuller et al. 2003; Adams et al. 2008…one identifies a stabilization point at 22% mortality, the other at 29% mortality…we used non-harvest mortality of 14% and combined it with the harvest rate estimate by the quota to get the stabilization rate… in a nutshell [that’s] how we get from the quota to a stabilization level.” Here, the authors note there are two to three additional published estimates as reviewed in the main text [2].

Following the above quotation, an NRB member asked about the two different estimates (1034 and 1195) of the wolf population that we review in main text and below in SM 1 Figure 1 legend. The DNR favored the higher estimate in response. The NRB member who posed the question (G. Kazmierski) indicated a personal preference for a higher quota but moved to approve the quota as follows [2].

G. Kazmierski moved, “… the NRB approve a quota of 200 non-reservation wolves and a permit number of 20 times the quota with a directive to the Department that no zone will be closed prior to the end of the season on February 28^th^ unless the full amount of harvest for that zone has been reached.” That motion passed unanimously. The above directive seems to constrain the DNR’s discretion in the matter. Also, the NRB over-ruled the DNR request for ten times the permits and instead doubled the number of permits made available to hunters. When the NRB member who brought the motion, G. Kazmierski, justified doubling the permit availability, he stated “We have a very short window to reach those harvest goals and objectives… because there is such a short window to accomplish this harvest by the end of February, by upping the number of hunters in the field, it will give us a better shot at filling that quota.” [2]

Following a question about timing and process, the DNR staff Warnke opined they “We would have been more confident and more comfortable had we taken more time.” [2]

The NRB chair asked about tribal declaration under federal tribal treaty rights (see section on Ojibwe tribal governments below). The DNR administrator K. Warnke clarified that “The tribes have not made a declaration yet. So those quotas typically …always by law… will be adjusted when we account for tribal declaration by treaty rights.” Then Chair Prehn asked for expansion on that issue, stating “Zone 1 has a harvest objective of 62……no matter what negotiations do with the tribe… that zone will not be closed until the quota is met?”. Then a new speaker from DNR agreed with the chair’s question. It remains unclear if the tribal declaration reserving 82 wolves reduced the zonal quotas which summed to 200 during the above meeting (SM 1 Fig 1) or if the NRB directed the DNR to permit 200 wolves to be killed “no matter what-negotiations do with the tribes” [2].


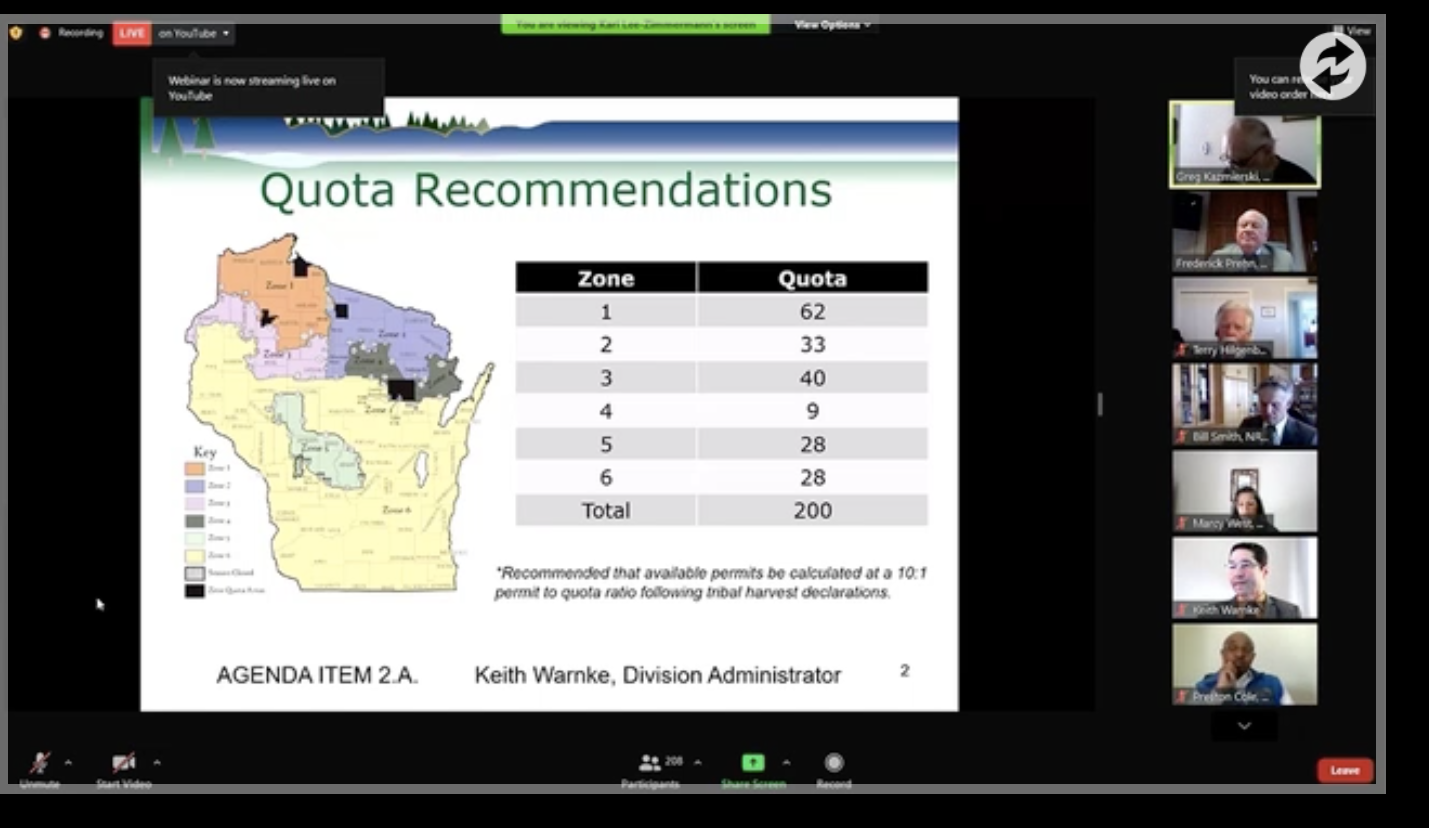


**SM 1 Figure 1.** Slide shown during the 15 February 2021 NRB meeting to vote on the DNR’s recommended quota, permit availability, and zonal quotas [2]

An NRB member asked about carcass collection for scientific purposes. The DNR responded they would not collect carcasses given the short time frame. “Typically the wolves that are harvested are not generally of breeding age, they are generally younger than breeding age wolves.“ The present authors are not aware of the science used to support that statement. We cited Stark [6] in the main text that seems inconsistent with the statement. Also see below for tribal report on carcass collection [2].

**Executive branch**

The DNR’s objectives for the February 2021 wolf-hunt were, “The quota's objective is to allow for a sustainable harvest that neither increases nor decreases the state's wolf population…The DNR is actively working to prepare for a fall 2021 wolf harvest season through a transparent and science-based process.” (<https://dnr.wisconsin.gov/topic/hunt/wolf/index.html> accessed 15 April 2021). We use the latest wolf kill total from 8 April 2021 [7] (SM Figure 2).


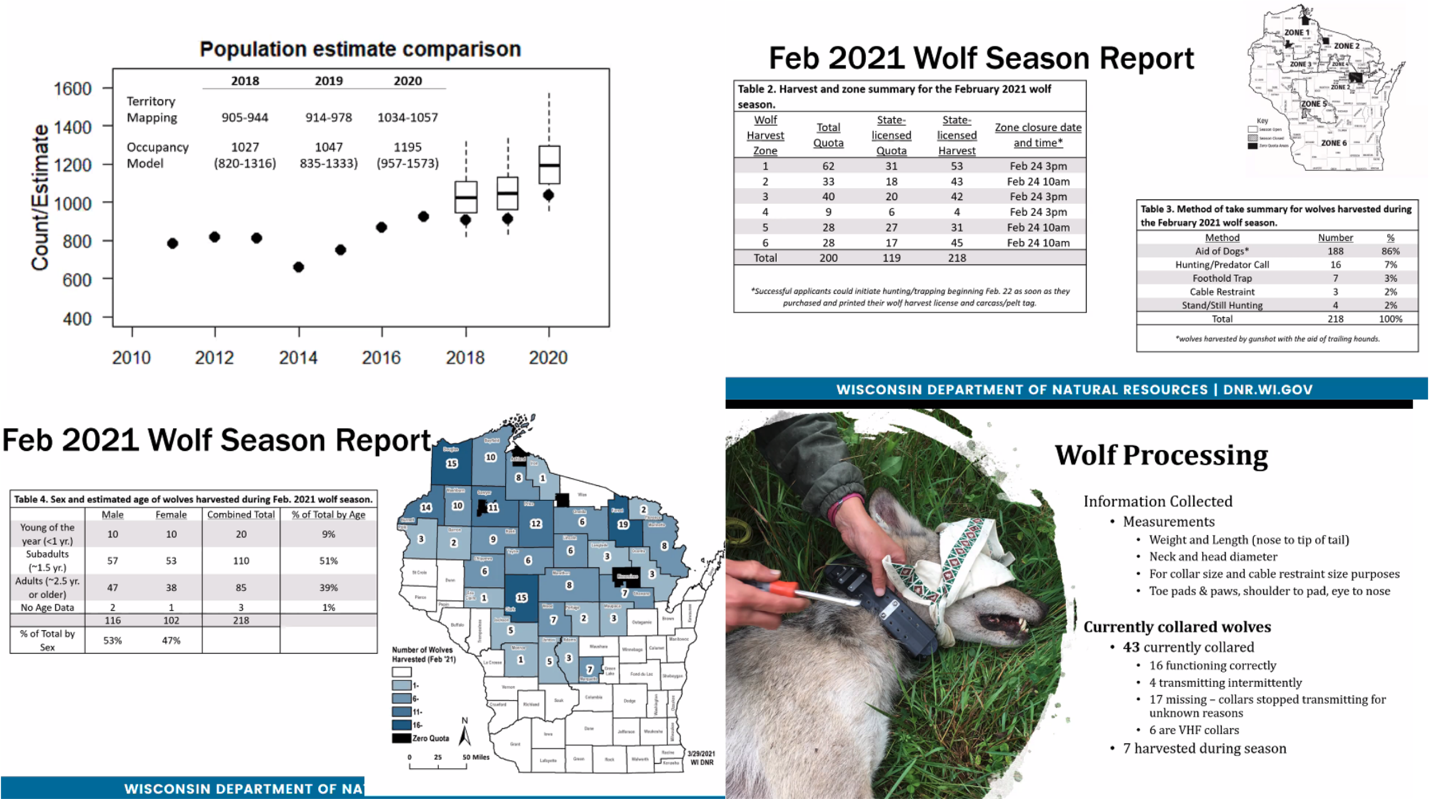


**SM 1 Figure 2.** Wisconsin Department of Natural Resources data presented on 8 April 2021 to the Wolf Harvest Committee and the public, showing (upper left) wolf population estimates, (upper right) hunter take by method, (lower left) hunter take by age and zone, (lower right) marked wolf fates. These are presented to substantiate population estimate and hunter take and not any other data because other potentially relevant data are unverified and ambiguous. There are three scientific reasons not to use the higher estimate in the main text: (1) The lower bound of the occupancy model lies below the estimate we use of 1034, yet it is presented graphically and in text without explaining if it is a 95% confidence interval or some other measure of uncertainty so the 1034 estimate is conservative; (2) the occupancy model has not undergone peer review as of writing; (3) the official state report on wolf population monitoring includes methods additional to the occupancy sample and appears to integrate multiple sources of information. This was a public meeting and presentation of slides <https://dnr.wisconsin.gov/calendar/meeting/42691#:~:text=Time%3A%2011%3A30%20a.m.%20%2D%202%3A30%20p.m.&text=The%202021%20Harvest%20Committee%20Meeting,providing%20Wolf%20Monitoring%20Program%20updates>, accessed 27 April 2021.

Regarding the assertion of science-based process, we summarize methods and timing of the hunt. A combination of environmental and anthropogenic factors, most notably the use of hounds and unprecedented issuance of permits relative to stated quotas, undermined the WDNR objective in its own words. For example, the Minneapolis Star Tribune reported,

“Fresh snowfall on Monday and Tuesday made wolf tracks easy to spot and captured their scent for trailing hounds to follow, said Randy Johnson, large carnivore specialist for the Wisconsin DNR. ‘This season was fairly unprecedented,’ Johnson said. ‘The use of dogs is a very efficient method of harvest.’ The Wisconsin DNR, which originally fought opening a season so soon after the animals were delisted, issued twice as many hunting permits than it had before for wolf hunts. It gave out 20 permits for every wolf it wanted harvested, rather than the more typical 10 per wolf. That's a decision that will be revisited, said Eric Lobner, director of the Wisconsin DNR's wildlife management program. ‘It's important for us to get more in line with what we normally issue for permits per quota,’ he said. The Wisconsin DNR planned for the hunt to last a week, but started shutting it down Tuesday afternoon [Feb 23, 2021] when hunters had reported enough kills to nearly fill the entire quota. Lobner and Johnson reiterated that the Upper Midwest's wolf population is robust and resilient. Before the hunt, the population was estimated to be around 1,200 wolves. The quota was set with the goal of keeping the population stable, Lobner said” [8], accessed 27 March 2021.

Also see the quotation from Warnke in the section on NRB above.

The state’s claim of no reduction in wolf population might merit consideration if sufficient pups were born after the wolf-hunting and poaching periods we studied. However, the timing of the wolf-hunt during the wolf mating season casts doubt on the relative success of wolf reproduction in 2021 because of the unprecedented February wolf-hunt. For example, a landscape with long-distance visibility enhanced by snow cover (27.5 cm in wolf range in Rhinelander, Wisconsin, a record-breaking snowfall in February 2021 https://waow.com/2021/02/05/snow-reports-from-february-4th/, accessed 27 March 2021) would have increased the risk to breeders posed by hunters, snowmobiles, and hounds pursuing wolves. Breeding alpha males and females would have been particularly conspicuous due to their territorial marking and courtship behaviors that lead them to urinate in tandem on snow and show conspicuous patterns of behavior and ranging during mating [9]. Also, the DNR reported that >85% of wolves were killed by hunters who used hounds, yet several efforts to measure this population of hunters back in 2001 and 2005 found them few in number and above average in age relative to likely wolf-hunters [10-12]. These data and the report (above) that 86% of wolves were killed by hunters using hounds raise questions about the role of hounds in killing wolves, which is illegal (p.6 in <https://widnr.widen.net/s/g9mtwx6vzw/2021-wolf-regulations>, accessed 29 March 2021). Therefore, our estimates in the main text are conservative because we used model parameters from periods in Wisconsin when wolf-killing was primarily done by government agents in response to real or perceived livestock threats rather than public hunting seasons with novel methods. Few such ‘agency kills’ occurred in February [13]. Our estimates are also conservative because our model parameters for cryptic poaching come from Wisconsin’s historical patterns rather than more recent, much higher estimates of cryptic poaching in Mexican gray wolves, which also have higher certainty [14].

Finally, the repeated claims above for a scientific basis for the wolf-hunt are inconsistent with recent evidence. We have shown previously that the 1999 population goal of 350 wolves could not be based on science and the state population model from 1999–2018 omitted crucial information [15]/. Furthermore, the state systematically under-estimated mortality rates during its wolf-hunt planning [13, 16, 17]. It did so again on 15 February 2021 when it estimated nonharvest mortality at 14% (https://dnrmedia.wi.gov/main/Play/ccb5cf0361c5471e9cbc7c7a898cfc741d?catalog=9da0bb432fd448a69d86756192a62f1721 accessed 27 April 2021, see testimony by D. MacFarland). Deficits in science seem to be the rule rather than the exception for North American wildlife management agencies, given a review of 667 wildlife hunting management plans across North America found the majority of cases made scientific claims about population estimates, quotas, or sustainability of hunting without providing evidence [18, 19].

**Public**

As of 27 March 2021, we found 173 articles or opinions since 1 January 2021 using Google News search for “Wisconsin” and “wolf” and removing an unrelated homicide case. In opinion articles and other venues, members of the public have accused the state of several missteps in management. Yet, very few media reports make claims that can be evaluated scientifically. However, one such claim is that the high rate of wolf killing reflected a state under-estimate of the wolf population (<http://www.onwisconsinoutdoors.com/WisconsinBlog/Wolf-Hunt-Meets-Harvest-Goal> accessed 27 March 2021). That is a scientific claim we can address.

The efficiency of legal wolf-killing (kills per unit time) should reflect a combination of encounter rate, hunter effort as a product of numbers and time spent searching, successful kills per attempted kill, and wolf detection as a product of wolf numbers and probability of detection. Any increment in hunter search effort even preceding the wolf-hunt, or conspicuousness of wolves might change the expectation for legal wolf-killing efficiency without the need for a gross inaccuracy in wolf population estimates. Therefore, we find no evidence has been presented to doubt the state estimate of the wolf population, especially given its consistency to historical pack sizes (1034 wolves in 256 packs mirrors the 40-year average pack size in the state) [20].

**Co-sovereigns of the State: Tribal governments**

By federal treaty, most of Wisconsin’s wolf range is co-managed by co-sovereign tribal governments and the State of Wisconsin [21]. Ojibwe tribes of the region revere and protect the wolf as brother or companion in their creation story and for ecological reasons such as maintaining the health of white-tailed deer populations and the health of medicinal plants on which the tribes depend [22-24]. Therefore, the tribes have opposed wolf-hunting in the past and continue to do so [25].

"Midway through the hunt, when the tribal government co-sovereign jurisdictions requested wolf carcasses, the state asked hunters to volunteer carcasses and 20 were turned in. As of writing, those carcasses had not been turned over to the tribes” (P. David, Great Lakes Indian Fish & Wildlife Commission, email to the lead author on 21 April 2021). Therefore, data on the number of breeding wolves killed or data on illegal methods, such as hound bites, will never be available to the public or independent scientific scrutiny for more than 8% of the legally killed wolves.

P. David also reported “I discovered, and DNR confirmed, that they erroneously set the quota 28 wolves higher than the Adams model they were using would have indicated.  Not earth shaking by itself, but another contributing factor to likely pop decline arising from the hunt.”(email to the lead author on 20 April 2021).

**References**

1. Natural Resources Board. Request that the board take action to consider approval of a quota for a february 2021 wolf hunt in accordance with the circuit court order issued on february 11, 2021 in hunter nation et al. V. Wdnr, et al, civ. No. 2021-cv-31 (jefferson county). Madison, Wisconsin Department of Natural Resources. , 2021 Contract No.: 3DC01AE6-681A-457C-AD29-0CC96F975FDE.

2. Natural Resources Board. 15 february 2021 special meeting. In: Board NR, editor. 2021. p. 37 min, transcript and video available from authors.

3. Stepp C. Dnr's cathy stepp: Wolf management is an art as well as a science. The Cap Times. 2013.

4. WDNR. Wisconsin wolf management plan. Madison, WI: Wisconsin Department of Natural Resources; 1999.

5. WDNR. Wisconsin wolf management plan addendum 2006 and 2007. Madison: Wisconsin Department of Natural Resources, 2007 August 15, 2007. Report No.

6. Stark D, Erb J. 2012 minnesota wolf season report. Grand Rapids, MN: Minnesota Department of Natural Resources, 2013 5 July 2013. Report No.

7. DNR. Presentation by j. Price tack to wolf harvest committee 8 aprl 2021. In: Resources DoN, editor. Madison, WI2021.

8. Stanley G. As minnesota considers wolf hunt, wisconsin hunters blow past quotas

wisconsin hunters killed nearly double their wolf quota in a three-day season. Minneapolis Star Tribune. 2021 25 February 2021.

9. Wydeven AP, Wiedenhoeft JE, Schultz RL, Thiel RP, Boles SH, Heilhecker E, et al. Progress report of wolf population monitoring in wisconsin for the period october - march 2004. In: Resources WDoN, editor. Park Falls, Wisconsin PUB-ER- 2004: Wisconsin Department of Natural Resources; 2004.

10. Naughton-Treves L, Grossberg R, Treves A. Paying for tolerance: The impact of livestock depredation and compensation payments on rural citizens' attitudes toward wolves. Conserv Biol. 2003;17:1500-11.

11. Treves A, Kapp KJ, Macfarland DM. American black bear nuisance complaints and hunter take. Ursus. 2010;21(1):30–42.

12. Treves A, Martin KA. Hunters as stewards of wolves in wisconsin and the northern rocky mountains, USA. Society and Natural Resources. 2011;24(9):984-94.

13. Treves A, Langenberg JA, López-Bao JV, Rabenhorst MF. Gray wolf mortality patterns in wisconsin from 1979 to 2012. J Mammal. 2017;98(1):17-32.

14. Louchouarn NX, Santiago-Ávila FJ, Parsons DR, Treves A. Evaluating how lethal management affects poaching of mexican wolves Open Science. 2021;8 (registered report):200330.

15. Treves A, Paquet PC, Artelle KA, Cornman AM, Krofel M, Darimont CT. Transparency about values and assertions of fact in natural resource management. Frontiers in Conservation Science: Human-Wildlife Dynamics. 2021;2:631998.

16. Santiago-Ávila FJ, Chappell RJ, Treves A. Liberalizing the killing of endangered wolves was associated with more disappearances of collared individuals in wisconsin, USA. Scientific Reports. 2020;10:13881.

17. Treves A, Artelle KA, Darimont CT, Parsons DR. Mismeasured mortality: Correcting estimates of wolf poaching in the united states. J Mammal. 2017;98(5):1256–64.

18. Artelle KA, Reynolds JD, A. T, Walsh JC, C. PP, Darimont CT. Hallmarks of science missing from north american wildlife management. Science Advances 2018;4(3):eaao0167.

19. Artelle KA, Reynolds JD, A. T, Walsh JC, C. PP, Darimont CT. Distinguishing science from “fact by assertion” in natural resource management. Science Advances (eLetter). 2018;4(3):eaao0167.

20. Wydeven AP, Wiedenhoeft J, Schultz RN, Thiel RP, Jurewicz RR, Kohn B, et al. History, population growth and management of wolves in wisconsin. In: Wydeven AP, Van Deelen TR, Heske EJ, editors. Recovery of gray wolves in the great lakes region of the united states: An endangered species success story. New York: Springer; 2009. p. 87-106.

21. Sanders JD. Wolves, lone and pack: Ojibwe treaty rights and the wisconsin wolf hunt. Wisconsin Law Review. 2013;2013:1263-94.

22. David P. Ma’iingan and the ojibwe. In: Wydeven AP, Van Deelen TR, Heske EJ, editors. Recovery of gray wolves in the great lakes region of the united states: An endangered species success story. New York: Springer; 2009. p. 267-78.

23. Shelley VS, Treves A, Naughton-Treves L. Attitudes to wolves and wolf policy among ojibwe tribal members and non-tribal residents of wisconsin's wolf range. Human Dimensions of Wildlife. 2011;16:397–413.

24. Fergus AR. Building carnivore coexistence on anishinaabe land: Gold standard non-lethal deterrent research and relationship building between livestock farmers and the bad river band of the lake superior tribe of chippewa indians. Madison, WI: University of Wisconsin; 2020.

25. Fergus A, Hill L. Mashkiiziibii wildlife program. Mashkiiziibii ma’iingan (gray wolf) relationship plan (edition 2. In: Department. MNR, editor. Odanah, WI: Bad River Band of Lake Superior Tribe of Chippewa Indians; 2019.
