# Supplementary material for: Quantifying the effects of delisting wolves after the first state began lethal management
Source: PeerJ. 2021 Jul 5;9:e11666. doi: 10.7717/peerj.11666 (PMC8265384; doi:10.7717/peerj.11666)
Supplement: Supplemental Information 3 [file peerj-09-11666-s003.docx]

**Supplementary Material 2: Why a population model without nonlinear effects is conservative**

Non-linear responses such as density-dependence and compensatory mechanisms acting on population dynamics have sometimes been identified for some wolf populations in some periods. However, using a more complex model is not justified for several reasons.

The general population ecology literature predicts when density-dependence will not be observed and Wisconsin’s wolves met three of the criteria: Fowler [1] long ago reviewed many mammal populations, wherein a majority -- but not all – of wildlife populations show density-dependence. Similarly, Brook and Bradshaw [2] added to our understanding with modeling of 1198 populations and explained why many conditions would prevent or obscure a density-dependent population dynamic for long periods or forever. We now cite our work published this month that documents three of those conditions to explain why Wisconsin wolves did not show negative density-dependence from 1980-2012 [3]. Indeed, it seems unsurprising that the Wisconsin wolves did not show negative density-dependence given pack territoriality, minimal changes in density over time, and the change in census methods 2 times between 1980-2012 and another 2 times between 2013 and the present.

Secondly, Stenglein et al. [4] and Chapron & Treves [5] agree there was no detectable negative density-dependence on mortality (which is our main focus). Although there is no consensus on density-dependence on reproduction, the only study finding such [4] found negative density-dependence which would tend to decrease the survival of juveniles to November 2020, increasing the percent reduction in the population reported. Our omission of such is therefore conservative. Furthermore, we find the evidence for negative density-dependence on recruitment is unsubstantiated [6, 7]. Therefore, inclusion of negative density-dependence is not justified and would only add to our estimate of percent reduction.

Some readers might wonder if there were compensatory effects on vital rates that might have arisen during our study period and might have led to an increase in the population or its rate of growth beyond the 3.8% average rate we began to model with. During our study period April 2020-April 2021, compensatory effects might surface as fewer deaths from other causes because hunters killed wolves, more immigration because of vacancies created by hunting, or more births or higher recruitment rates because of space or resources freed up by the death of adults during the hunt. However the hunt occurred at the end of February 2021, so compensatory effects of the hunt, if any, would emerge afterwards. Our modeling concerns April 2020-April 2021. Claims about compensatory effects that might recover lost population have to show evidence for such effects AFTER the hunt.

Therefore, our simple population model, based on average growth rates of previous years, already incorporates potential structural non-linear effects to predict the population by November 2020 and then we begin deducting the additional human-caused deaths that began with federal delisting. The cumulative incidence functions we describe below already incorporate non-linear effects on mortality but research has proven those effects are depensatory not compensatory [8, 9], which is consistent with population-level analyses showing super-additive mortality [10] and depensatory population decline [5, 6, 11-13].

Regarding the inferences we made about depensatory effects, below we explain how the inferences about cryptic poaching were made with confidence.

We used time-to-event analyses on radio-collared wolves in two populations (and two independent datasets) to evaluate the changes in survival of wolves over time as policies changed. The policies in question were reductions in ESA protections interspersed with periods of stricter protection. We showed that disappearances of radio-collared wolves increased substantially during periods with reduced ESA protections, estimated at 19% in Wisconsin’s less intensively monitored population [8] and 121% in the Mexican gray wolf population monitored five-ten times more intensively by several measures [9]. But it is not only the association with 6 changes in policy that allow us to estimate cryptic poaching.

We also examined mechanical failures of collars and migration. There are only three known outcomes for wolves with radio-collars that are lost to monitoring by radio-telemetry. First the transmitter may undergo mechanical or battery failure. Second, the collared animal may migrate out of range of telemetry. Third, the animal may die and its transmitter be destroyed by people.

There is no known mechanism by which policy change can cause mechanical or battery failure but moreover, disappearances of radio-collared wolves occur several hundred to 1000s of days earlier than expected for the life of radio-collars judging from the average life of collars in wolves that die from natural deaths, as we have shown for a number of populations of wolves [14-16].

As for migration, a radio-collared wolf must leave the state not simply shift range to be lost to monitoring. For Mexican gray wolves, the intensive monitoring once per week or twice per week and assiduous search for missing wolves did not reveal more than perhaps one case in >400 of a wolf with radio-collar migrating so far and being lost to contact, i.e., even migrants are often recovered [9]. A red wolf study with >500 radio-collared wolves reported the same pattern [14]. Although Wisconsin wolves were not monitored as intensively then or now, our prior work showed migration was seven times more frequent into Wisconsin from Michigan than the converse [15], and moreover we used the more conservative cumulative incidence curves from Wisconsin rather than the much more dramatic 121% increase in LTF from Mexican gray wolves. Note emigrants are nonetheless lost to Wisconsin’s wolf population making emigration an unsatisfactory rebuttal of our estimate of population decline.

We consider it vanishingly rare that a radio-collared wolf died of a non—poaching cause in a medium or substrate (e.g., salt water or underground) that destroyed the transmitter soon after death, although a case of poaching and dumping in saltwater is known [14].

The only remaining possible cause of disappearance of a monitored wolf is human manipulation. A great deal of social scientific data also support the willingness and intention to poach wolves in Wisconsin and beyond [17-21]. That evidence was summarized in Science [22].

**References cited**

1. Fowler, C.W., *A Review of Density Dependence in Populations of Large Mammals*, in *Current Mammalogy*, G. H.H., Editor. 1987, Springer: Boston. p. 401-441

2. Brook, B. and C. Bradshaw, *Strength of evidence for density dependence in abundance time series of 1198 species.* Ecology, 2006. **87**: p. 1445-1451.

3. Treves, A., P.C. Paquet, K.A. Artelle, A.M. Cornman, M. Krofel, and C.T. Darimont, *Transparency about values and assertions of fact in natural resource management.* Frontiers in Conservation Science: Human-Wildlife Dynamics, 2021. **2**: p. 631998. DOI: 10.3389/fcosc.2021.631998.

4. Stenglein, J.L., J. Zhu, M.K. Clayton, and T.R. Van Deelen, *Are the numbers adding up? Exploiting discrepancies among complementary population models.* Ecology and Evolution, 2015. **5**(2): p. 368-376.

5. Chapron, G. and A. Treves, *Blood does not buy goodwill: allowing culling increases poaching of a large carnivore.* Proceedings of the Royal Society B, 2016. **283**(1830): p. 20152939. <http://dx.doi.org/10.1098/rspb.2015.2939>

6. Chapron, G. and A. Treves, *Reply to comment by Pepin et al. 2017.* Proceedings of the Royal Society B, 2017. **2016257**(1851): p. 20162571. <http://dx.doi.org/10.1098/rspb.2015.2939>

7. Chapron, G. and A. Treves, *Reply to comments by Olson et al. 2017 and Stien 2017.* Proceedings of the Royal Society B, 2017. **284**(1867): p. 20171743.

8. Santiago-Ávila, F.J., R.J. Chappell, and A. Treves, *Liberalizing the killing of endangered wolves was associated with more disappearances of collared individuals in Wisconsin, USA.* Scientific Reports, 2020. **10**: p. 13881. /10.1038. | <https://doi.org/10.1038/s41598-020-70837-x>.

9. Louchouarn, N.X., F.J. Santiago-Ávila, D.R. Parsons, and A. Treves, *Evaluating how lethal management affects poaching of Mexican wolves* Open Science, 2021. **8 (registered report)**: p. 200330. <https://doi.org/10.1098/rsos.200330>.

10. Creel, S. and J.J. Rotella, *Meta-analysis of relationships between human offtake, total mortality and population dynamics of gray wolves (Canis lupus).* PLoS ONE, 2010. **5**(9): p. 1-7.

11. Vucetich, J.A., *Appendix: The influence of anthropogenic mortality on wolf population dynamics with special reference to Creel and Rotella (2010) and Gude et al. (2011) in the Final peer review of four documents amending and clarifying the Wyoming gray wolf management plan.* Federal Register, 2012. **50**: p. 78-95. <https://www.federalregister.gov/documents/2012/05/01/2012-10407/endangered-and-threatened-wildlife-and-plants-removal-of-the-gray-wolf-in-wyoming-from-the-federal>.

12. Chapron, G. and A. Treves, *Correction to ‘Blood does not buy goodwill: allowing culling increases poaching of a large carnivore’.* Proceedings of the Royal Society B, 2016. **Volume 283**(1845): p. 20162577.

13. Chapron, G., Y. Epstein, A. Trouwborst, and J.V. López-Bao, *Bolster legal boundaries to stay within planetary boundaries.* Nature Ecology & Evolution, 2017. **1**(0086): p. doi:10.1038/s41559-017-0086.

14. Agan, S.W., A. Treves, and L. Willey, *Wild red wolf Canis rufus poaching risk.* bioRxiv, 2020. <https://doi.org/10.1101/2020.12.08.416032>.

15. Treves, A., J.A. Langenberg, J.V. López-Bao, and M.F. Rabenhorst, *Gray wolf mortality patterns in Wisconsin from 1979 to 2012.* Journal of Mammalogy, 2017. **98**(1): p. 17-32. DOI:10.1093/jmammal/gyw145.

16. Treves, A., K.A. Artelle, C.T. Darimont, and D.R. Parsons, *Mismeasured mortality: correcting estimates of wolf poaching in the United States.* Journal of Mammalogy, 2017. **98**(5): p. 1256–1264. I:10.1093/jmammal/gyx052 <https://doi.org/10.1093/jmammal/gyx052>.

17. Browne-Nuñez, C., A. Treves, D. Macfarland, Z. Voyles, and C. Turng, *Tolerance of wolves in Wisconsin: A mixed-methods examination of policy effects on attitudes and behavioral inclinations.* Biological Conservation, 2015. **189**: p. 59–71.

18. Hogberg, J., A. Treves, B. Shaw, and L. Naughton-Treves, *Changes in attitudes toward wolves before and after an inaugural public hunting and trapping season: early evidence from Wisconsin’s wolf range.* Environmental Conservation, 2015. **43**(1): p. 45-55.

19. Naughton-Treves, L., R. Grossberg, and A. Treves, *Paying for tolerance: The impact of livestock depredation and compensation payments on rural citizens' attitudes toward wolves.* Conservation Biology, 2003. **17**: p. 1500-1511.

20. Treves, A. and K.A. Martin, *Hunters as stewards of wolves in Wisconsin and the Northern Rocky Mountains, USA.* Society and Natural Resources, 2011. **24**(9): p. 984-994.

21. Treves, A., L. Naughton-Treves, and V.S. Shelley, *Longitudinal analysis of attitudes toward wolves.* Conservation Biology, 2013. **27**: p. 315–323.

22. Treves, A. and J.T. Bruskotter, *Tolerance for predatory wildlife.* Science, 2014. **344**(6183): p. 476-477.
